# Thermoelectric Sensor with CuI Supported on Rough Glass

**DOI:** 10.3390/nano14010105

**Published:** 2024-01-01

**Authors:** Gustavo Panama, Seung S. Lee

**Affiliations:** Department of Mechanical Engineering, Korea Advanced Institute of Science and Technology, Daejeon 34141, Republic of Korea; gustavsp@kaist.ac.kr

**Keywords:** thermoelectric sensor, copper iodide (CuI), cobalt antimonide (CoSb_3_), rough glass

## Abstract

Thermoelectric generators convert heat into a potential difference with arrays of p- and n-type materials, a process that allows thermal energy harvesting and temperature detection. Thermoelectric sensors have attracted interest in relation to the creation of temperature and combustible gas sensors due to their simple operation principle and self-powering ability. CuI is an efficient p-type thermoelectric material that can be readily produced from a Cu layer by an iodination method. However, the vapor iodination of Cu has the disadvantage of weak adhesion on a bare glass substrate due to stress caused by crystal growth, limiting microfabrication applications of this process. This work presents a rough soda-lime glass substrate with nanoscale cavities to support the growth of a CuI layer, showing good adhesion and enhanced thermoelectric sensitivity. A rough glass sample with nanocavities is developed by reactive ion etching of a photoresist-coated glass sample in which aggregates of carbon residuals and the accumulation of NaF catalyze variable etching rates to produce local isotropic etching and roughening. A thermoelectric sensor consists of 41 CuI/In-CoSb_3_ thermoelectric leg pairs with gold electrodes for electrical interconnection. A thermoelectric leg has a width of 25 μm, a length of 3 mm, and a thickness of 1 μm. The thermoelectric response results in an open-circuit voltage of 13.7 mV/K on rough glass and 0.9 mV/K on bare glass under ambient conditions. Rough glass provides good mechanical interlocking and introduces important variations of the crystallinity and composition in the supported thermoelectric layers, leading to enhanced thermopower.

## 1. Introduction

Thermoelectric generators convert heat into a potential difference based on the Seebeck effect [1]. Thermoelectric p–n modules produce electric energy from a thermal gradient across arrays of n- and p-type materials, enabling power generation and self-powered sensor applications [2]. In general, the voltage generated is proportional to both the thermal gradient and the number of thermoelectric leg pairs. A reduced voltage signal is ineffective for applications showing low heat emission levels, such as combustible gas sensors and human body heat, among others [3,4,5,6]. A good voltage signal can be generated from heat from thermoelectric materials with enhanced sensitivity, which can be effective for micro thermoelectric devices operating under a low thermal gradient.

CuI is an efficient p-type inorganic semiconductor and thermoelectric material due to its high electrical conductivity [7,8]. CuI is readily prepared from a Cu layer by vapor (g), liquid (l), or solid (s) iodination at low temperatures [9,10,11]. Cu is converted into CuI by the redox reaction of 2Cu + I_2_ → 2CuI [12]. However, the iodination of Cu/glass produces a CuI layer that has poor adhesion, representing a disadvantage for microelectronic applications involving photolithography and wet processes [13]. The interfacial stability of CuI/glass is likely compromised owing to lattice expansion and stress caused by crystal growth, which increases the layer thickness by three to five times [10,12].

The Seebeck coefficient or thermopower (S) increase for thermoelectric materials shows reduced charge carrier concentrations, an outcome directly related to electric conductivity [14,15]. Electrical conductivity can be reduced for a thermoelectric layer with numerous interfacial defects [16,17]. Interfacial defects can be beneficial to tailor an enhanced potential difference in a thermoelectric layer due to charge carrier traps and grain boundaries that reduce the facile charge flow, which implies an increment of the electrical resistance. Reduced electrical conductivity is suitable for thermoelectric sensors that operate under an open-circuit configuration, which is the opposite for thermoelectric generators that require reduced charge scattering and an enhanced charge flow [18,19].

CuI has low electronic and lattice thermal conductivity rates due to the presence of heavy iodide atoms and the grain boundaries of a polycrystalline layer that slows down the phonon group velocity and enhances carrier screening, respectively [16,20,21]. CuI can show enhanced thermovoltage responses owing to the higher density of the grain boundaries caused by rough glass with nanocavities, producing a CuI layer with both reduced electrical conductivity and enhanced adhesion [22]. The enhanced adhesion can be attributed to the mechanical interlocking of the CuI layer growing into the nanocavities. Thus, a rough glass surface with nanocavities is likely to stabilize CuI and improve its thermopower.

Glass surfaces can be readily modified by various methods, including wet or dry etching [23]. The surface roughness increases during the reactive ion etching of glass with a very thin metal layer, which results in isolated metal islands that inhibit the effects of etching reactions [24]. However, conventional surface roughening is ineffective if attempting to anchor a layer with weak adhesion. A photoresist layer can be employed as a seed layer in dry etching to enable roughening and the creation of nanocavities on the coated region due to the residual porous layer arising from the erosion of the photoresist and glass. A porous residual layer produces a micromask effect, leading to variable etching rates and an isotropic-like etching profile.

**Figure 1:**
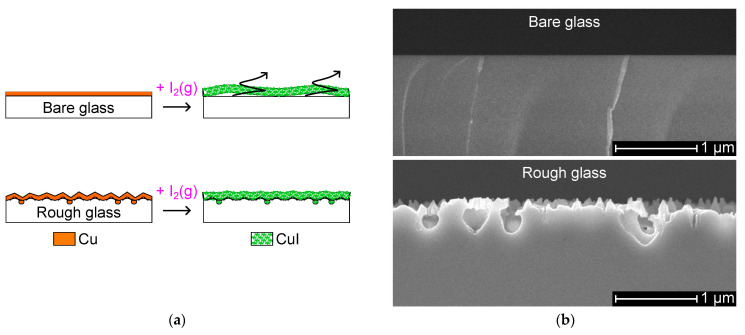
Process of creating the CuI layer: (**a**) Conversion of Cu into CuI by vapor iodination on bare and rough glass. (**b**) Cross-sectional view of bare and rough soda-lime glass substrates.

This work proposes a rough soda-lime glass substrate to support the growth of thick CuI from vapor iodination of a Cu layer in the air by interlocking into nanocavities, as shown in Figure 1a. Conversely, a CuI layer on bare glass is likely to detach due to reduced adhesion strength. Figure 1b shows a rough glass surface prepared by a reactive ion etching (RIE) treatment of bare glass coated with a photoresist seed layer [25]. The photoresist layer causes roughening of the glass surface due to decomposition under reactive plasma, which produces a micromask. A CuI/In-CoSb_3_ p–n thermoelectric sensor is employed in a catalytic thermoelectric hydrogen sensor and a touch/exhalation detector.

## 2. Materials and Methods

### 2.1. Device

The thermoelectric sensor is composed of n-type and p-type thermoelectric materials which are connected electrically in a series on metal electrodes and thermally in a parallel design [1]. Enhanced sensitivity is obtained by the synergetic effect of enhanced surface roughness of the soda-lime glass substrate and the use of good thermoelectric materials for low-temperature applications (<400 K). Soda-lime glass substrates are attractive for thermoelectric devices due to their low thermal conductivity coefficient (1.12 W/m∙K, 373 K), resulting in good thermal isolation [26]. Figure 1b shows a cross-sectional view of a reference bare glass sample and a rough glass sample showing mean surface roughness values (Ra) of 1 nm and 10 nm, respectively.

**Figure 2:**
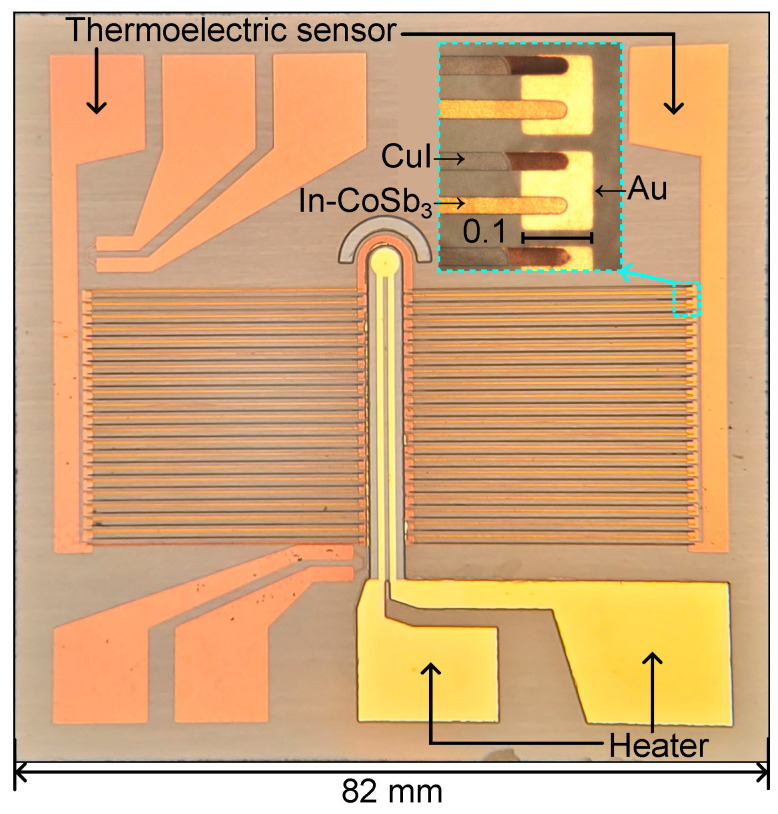
Elements composing the CuI/In-CoSb_3_ thermoelectric sensor on rough glass.

The CuI/In-CoSb_3_ thermoelectric sensor is developed using bare and rough soda-lime glass substrate unless otherwise indicated. A rough glass substrate produces abundant cracks and grain boundaries in the supported layers with enhanced adhesion due to material growth into nanocavities. Figure 2 shows the proposed thermoelectric sensor, which consists of 41 CuI/In-CoSb_3_ thermoelectric pairs of a width of 25 μm, a length of 3 mm, and a thickness of 1 μm connected electrically in series on gold electrodes. Thermoelectric pairs are arranged thermally parallel to a 100 Ω gold heater. The heater is used to produce a temperature gradient across the thermoelectric pairs.

### 2.2. Fabrication

**Figure 3:**
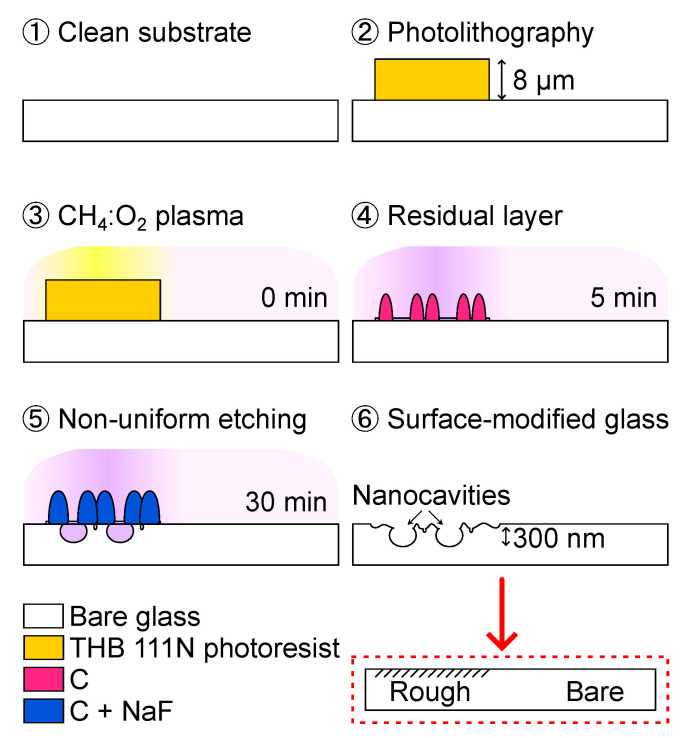
Formation mechanism of a rough soda-lime glass substrate with nanocavities.

The fabrication of a thermoelectric sensor requires a rough soda-lime substrate to support a CuI layer produced by vapor iodination. Surface modification of a glass substrate is important to increase the surface roughness and develop nanocavities for mechanical interlocking of the CuI layer. Figure 3 presents the formation mechanism of a rough glass substrate with nanocavities, with this mechanism also described below. Note: the sample is rinsed with deionized water and dried with nitrogen after each step of the wet process, i.e., cleaning, development, and lift-off.

1. Clean a soda-lime glass substrate in a piranha solution with a volume ratio of 3:1 for H_2_SO_4_ (96%):H_2_O_2_ (30%) for 15 min.
2. Spin-coat a negative photoresist (THB 111N, JSR Corporation, Tokyo, Japan) at 2000 rpm for 30 s. Soft-bake in a convection oven at 393 K for 5 min. Transfer the pattern from the photomask using a mask aligner in vacuum-contact mode. Expose the sample to 365 nm UV light and deliver a total dose of 300 mJ/cm^2^. Develop the sample in a developer solution (AZ 300 MIF, Merck Electronic Materials Co., Ltd., Anseong, Republic of Korea) while gently shaking for 6 min. Hard-bake the sample on a hotplate for 10 min. Note: the glass with a photoresist layer undergoes a surface modification, and the photoresist layer is 8 μm thick.
3. Use an RIE system (URL-100, ULTECH Co., Ltd., Daegu, Republic of Korea) for sample treatment at 200 mTorr with 10 sccm CF_4_ and 30 sccm O_2_. Plasma is produced by a 13.56 MHz RF power generator at 100 W. The sample chuck is continuously cooled to 288 K with a chiller.
4. Carbon residuals can form on the photoresist-coated areas after RIE treatment for 5 min, initiating non-uniform etching reactions. The plasma glow color changes from white to light purple.
5. Nanocavities are formed due to local masking effects caused by carbon residuals and the aggregation of less volatile components from the etching of the glass surface.
6. Clean the RIE-treated sample in an ultrasonic bath with a photoresist stripper (STR-F, Hana Corporation Co., Ltd., Seongnam, Republic of Korea) for 1 h at 333 K. An arrow indicates the schematic for rough and bare glass used in the next section.

**Figure 4:**
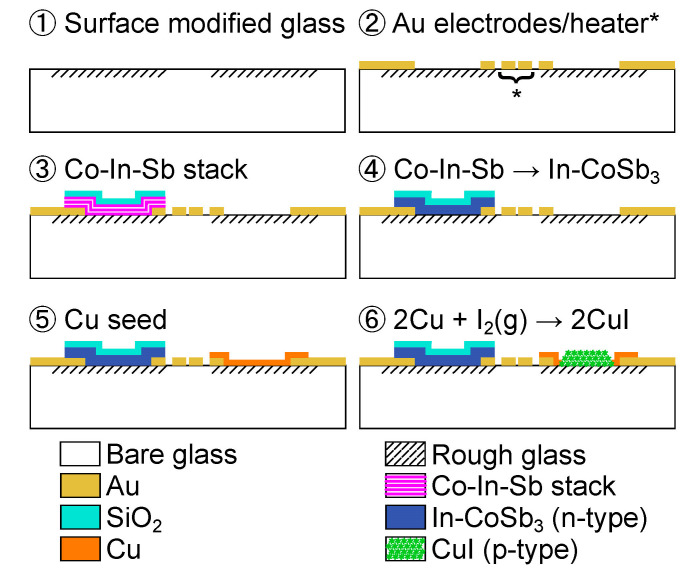
Fabrication process of the CuI/In-CoSb_3_ thermoelectric sensor. The heater location is shown by the asterisk (*).

The layers composing the thermoelectric sensor are developed by a microfabrication process using conventional photolithography. Figure 4 shows the fabrication process of the CuI/In-CoSb_3_ thermoelectric sensor, in which the lift-off technique is used in processes #2, 3, and 5:

1. Prepare a surface-modified glass according to the procedure in Figure 3.
2. Pattern a sacrificial layer for lift-off of the Au electrode and heater with an AZ-nLOF-2070 photoresist 5 μm thick (Clariant Corporation, Somerville, NJ, USA). Deposit 70 nm Au (2.5 Å/s) on 30 nm of Cr (2.0 Å/s) by e-beam evaporation at 6 × 10^−6^ Torr. The heater location is shown by the asterisk (*).
3. Pattern a photoresist layer for lift-off of the Co-In-Sb stack. Deposit seven cycles of a Co-In-Sb stack composed of 90 nm Sb (4.0 Å/s), 5 nm In (0.4 Å/s), and 9 nm Co (1.0 Å/s) on a 30 nm Cr adhesion layer. Deposit 250 nm SiO_2_ (1.5 Å/s) as a capping layer.
4. Convert the Co-In-Sb stack into an In-doped CoSb_3_ layer by thermal annealing at 573 K for 20 min under ambient conditions.
5. Pattern a photoresist layer for the lift-off of Cu. Deposit 160 nm Cu (4.0 Å/s) on a 3 nm Cr adhesion layer.
6. Pattern a masking layer to expose only Cu on the textured glass with an AZ-10XT photoresist (520 cP) 7 μm thick (Clariant Corporation, Somerville, NJ, USA). Expose to iodine vapor in a sealed beaker on a hot plate at 373 K for 3 min under ambient conditions. Remove the photoresist.

### 2.3. Measurement Setup

**Figure 5:**
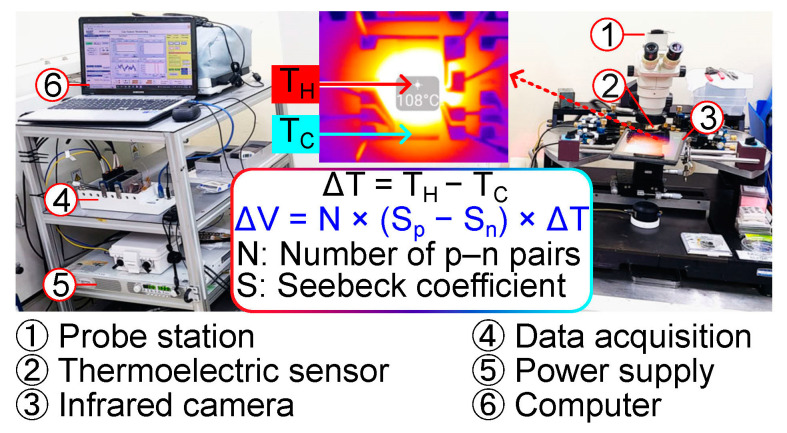
Measurement setup for thermoelectric sensor response.

The thermoelectric response is measured in a probe station equipped with an infrared camera (CompactPRO, Seek Thermal Inc., Santa Barbara, CA, USA; sensitivity: 1 K), a data acquisition system, and a power supply, as displayed in Figure 5. The infrared camera is calibrated and shows a measurement deviation of 0.2 ± 1.6 K compared to a fine wire thermocouple, which allows accurate monitoring of the point temperature [27]. The temperatures of the cold and hot regions are recorded in video format at 15 frames per second, and the temperature data are obtained by image post-processing in MATLAB 2023b. The temperature difference (ΔT = T_H_ − T_C_) is obtained from two successive Joule heating tests with the incorporated heater. Point temperature is monitored on the interconnection electrodes at the center (T_H_) and border (T_C_) of the thermoelectric sensor, as indicated in a thermal image in Figure 5. The voltage output is recorded at 10 Hz. The thermal gradient is produced with the incorporated heater while applying various voltages.

## 3. Results

### 3.1. Characterization of the Glass Substrate

**Figure 6:**
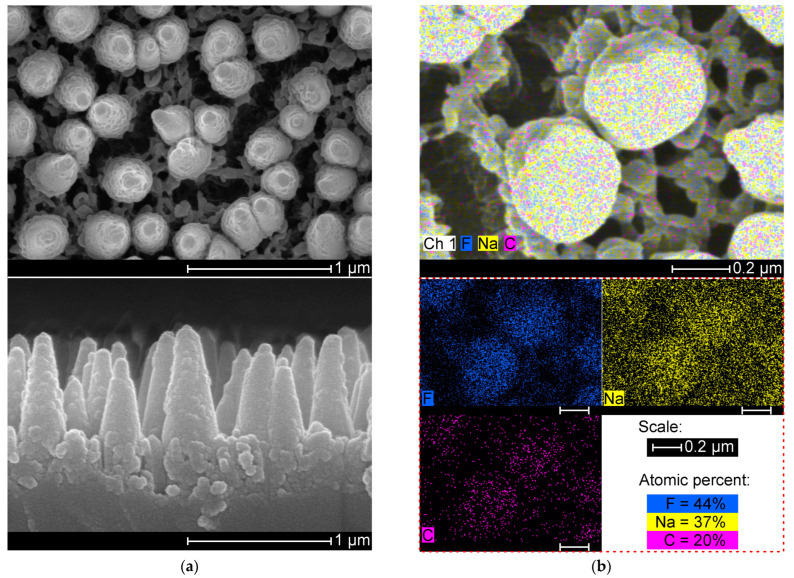
Characterization of the residual layer: (**a**) Scanning electron images showing the surface morphology (**top**) and cross-sectional view (**bottom**) of a residual layer formed during the reactive ion plasma treatment on photoresist-coated glass. (**b**) Elemental mapping by energy dispersive X-ray spectroscopy of a residual layer.

The glass substrate was examined after the RIE treatment. Scanning electron microscope (SEM) observations reveal a residual layer of cone-shaped posts on the RIE-treated glass surface. The posts have similar dimensions and are randomly distributed in a porous ground layer, as depicted in Figure 6a. Figure 6b shows the results of a composition analysis of the residual layer by X-ray energy dispersive spectroscopy. F, Na, and C are mainly detected at corresponding concentrations of 44, 37 and 20%.

**Figure 7:**
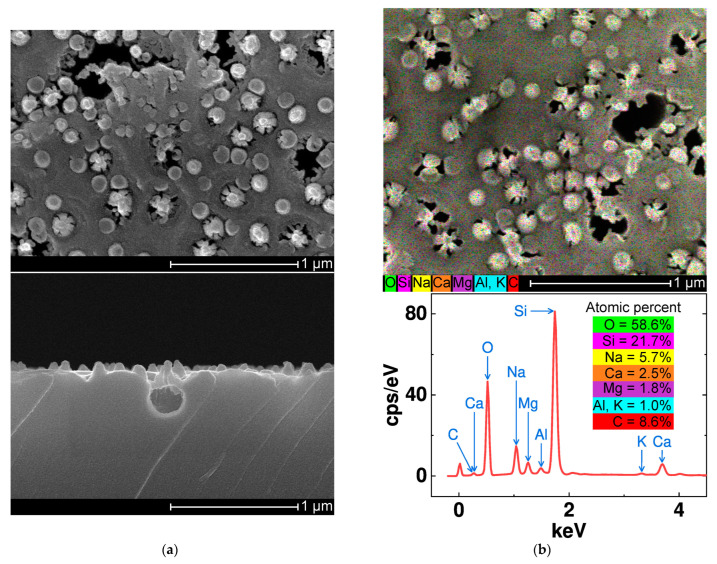
Characterization of a rough glass specimen: (**a**) Scanning electron images showing the surface morphology (**left**) and cross-sectional view (**right**) of the rough glass specimen. (**b**) Spectrum obtained by energy dispersive X-ray spectroscopy of the rough glass specimen.

The RIE-treated glass substrate was evaluated after a cleaning step. Figure 7a shows the surface morphology and a cross-sectional view of an RIE-treated glass sample. The resulting glass surface exhibits irregular erosion, nanoscale cavities (Φ = 100–300 nm), and cone-shaped posts (h = 70 nm, Φ = 100 nm) that are randomly distributed on a bumpy surface. Figure 7b displays the EDS spectrum of a rough glass surface with the chemical composition of typical soda-lime glass, and its constituent elements are indicated with arrows. The rough glass surface is free of hard polymer residual debris, and mapping of the carbon concentration yields the default detection range via standard SEM.

**Figure 8:**
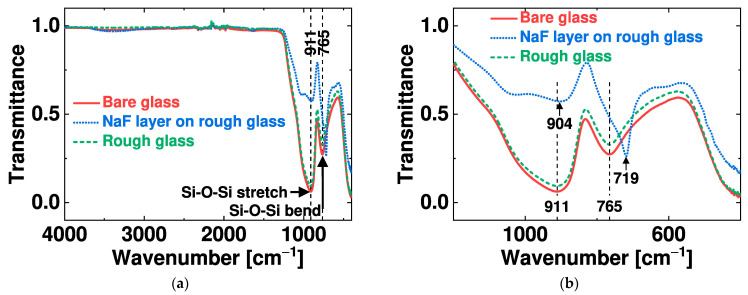
Surface analysis of glass specimens by Fourier transform infrared spectrometry: (**a**) Transmission spectra of bare glass, glass with a residual layer, and rough glass. (**b**) Expanded transmission spectra at low wavenumbers.

The organic residuals on the rough glass substrate were analyzed by Fourier transform infrared (FTIR) spectrometry. Figure 8a presents the transmission spectra of three glass specimens: bare glass (control), glass with a residual layer, and rough glass. The residual layer that forms during the RIE treatment is composed of NaF and carbon, as shown in Figure 6b. The presence of F in the silicate networks shifts the stretching and bending vibrational modes of Si-O-Si towards lower wavenumbers, as shown in Figure 8b [28]. A shift to a lower wavenumber suggests degradation of the silicate structure, resulting in reduced energy of the Si–O bonds attributable to the residual layer weakly bonded onto the glass surface [29]. A cleaned rough glass sample shows a transmission spectrum similar to that of bare glass, which suggests a lack of hard organic residuals or fluorinated carbon [30].

### 3.2. Characterization of Thermoelectric Layers

**Figure 9:**
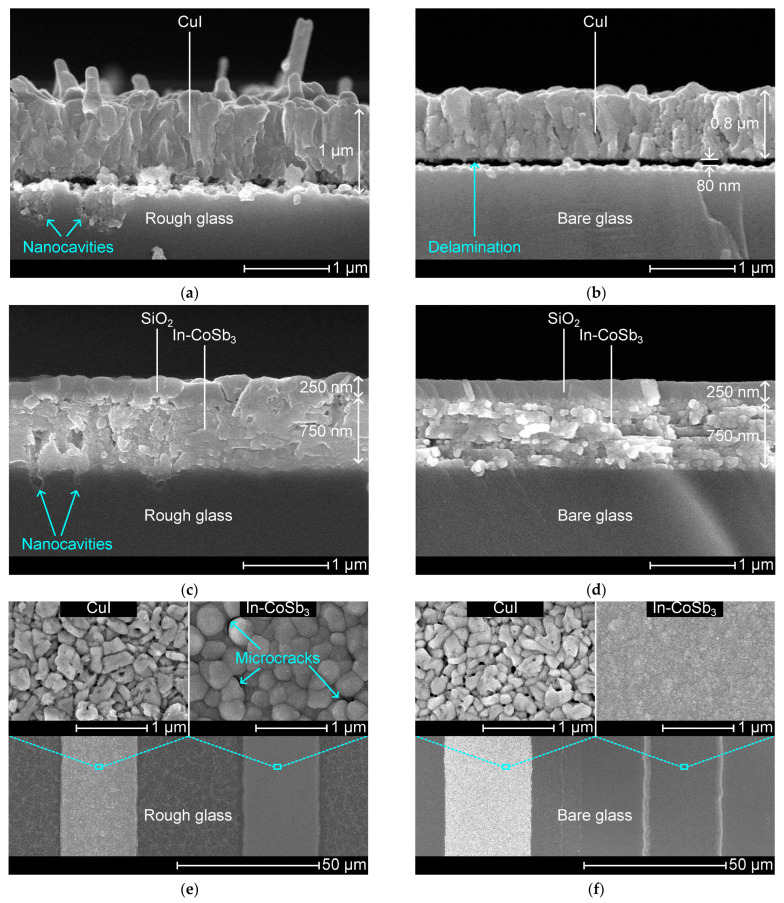
Scanning electron microscope images of thermoelectric layers. Cross-sectional view of CuI on (**a**) rough and (**b**) bare glass. Cross-sectional view of In-CoSb_3_ on (**c**) rough and (**d**) bare glass. Surface morphology of CuI (left) and In-CoSb_3_ (right) on (**e**) rough and (**f**) bare glass.

The thermoelectric layers on the rough and bare glass specimens are examined by SEM imagery. Figure 9a,b displays a cross-sectional view of a CuI layer on the rough and bare glass specimens, respectively. CuI on rough glass is ca. 1 μm thick, and CuI on bare glass is ca. 0.8 μm thick, where the thicker CuI layer can be attributed to the relatively high amount of Cu seed material per unit length due to the enhanced surface area. The rough glass produces a rougher CuI layer than the bare glass. Also, CuI on the rough glass exhibits nanorod-like structures on the surface. It was also noted that CuI is anchored in the nanocavities on the rough glass, whereas CuI shows a delamination gap on the bare glass.

Moreover, Figure 9c,d shows a cross-sectional view of In-CoSb_3_ on both types of glass. The In-CoSb_3_ layer is 750 nm thick and is capped with a 250 nm-thick SiO_2_ layer. In-CoSb_3_ shows good interfacial adhesion on both glass specimens. SiO_2_ on the rough glass exhibits microcracks that expose the underneath layers to the atmosphere, whereas SiO_2_ on the bare glass is free of defects, providing the ideal passivation of In-CoSb_3_. The rough glass produces a bumpy In-CoSb_3_ layer with greater surface roughness.

The surface morphologies of CuI and In-CoSb_3_ on the rough and bare glass are shown in Figure 9e,f, respectively. Expanded images reveal a CuI layer with similar grain size and morphology on both glass specimens. CuI shows numerous interfacial defects and voids. The In-CoSb_3_ layer exhibits a coarse- and fine-grained surface in the rough and bare glass cases, respectively. An expanded image of In-CoSb_3_ confirms that noticeable microcracks developed on the rough glass.

**Figure 10:**
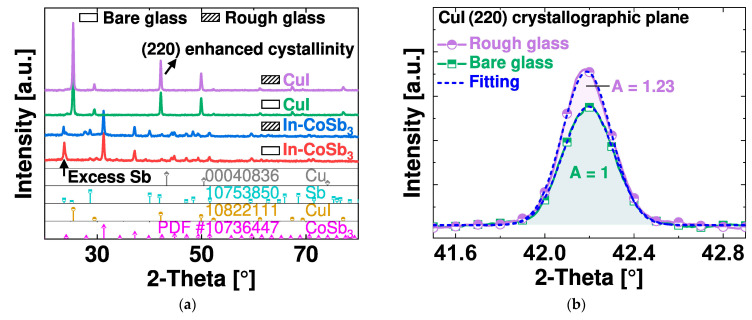
X-ray diffraction peaks of thermoelectric layers: (**a**) In-CoSb_3_ and CuI on bare and rough glass. (**b**) Expanded spectra of the (220) crystallographic plane of CuI.

X-ray diffraction (XRD) was used to reveal the crystal properties of the CuI and In-CoSb_3_, as shown in Figure 10a. The Cu layer is treated with iodine vapor to obtain polycrystalline CuI [11,31]. The Co-In-Sb stack is thermally converted into a layer of In-CoSb_3_, which shows a polycrystalline structure with multiple diffraction peaks [32,33]. Also, a noticeable peak appears at a 2θ of 23.7º for In-CoSb_3_ on bare glass attributable to crystal domains with excess Sb. The crystallographic patterns reveal that the target thermoelectric materials are properly synthesized. Figure 10b shows diffractograms of the (220) crystallographic plane of CuI. Among the various dominant crystallographic planes of CuI, a noticeable intensity difference is observed for (220), indicative of preferential crystal growth along the normal direction of the surface of the rough glass.

### 3.3. Test Results

**Figure 11:**
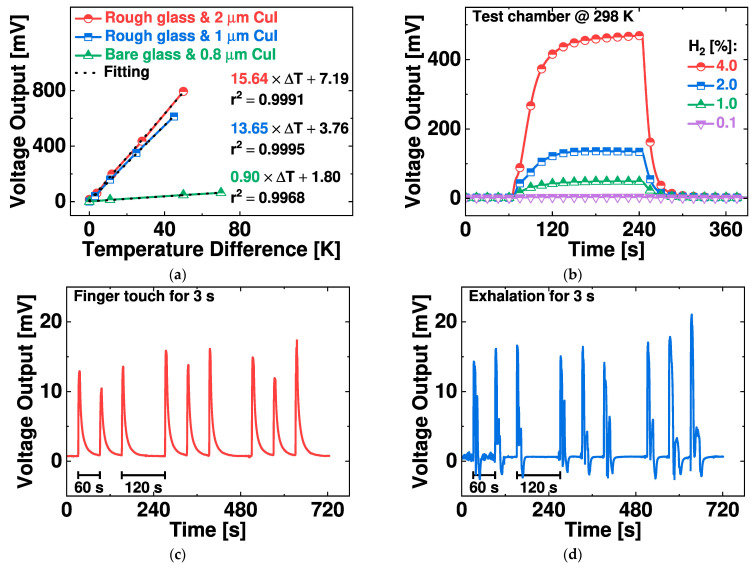
Test results of the CuI/In-CoSb_3_ thermoelectric sensor: (**a**) Thermoelectric response. (**b**) Hydrogen-sensing response after coating with Pt/TiO_2_ catalytic ink on the heater region. Voltage output response to (**c**) a finger touch for 3 s and (**d**) exhalation for 3 s at the center of the device.

In-CoSb_3_/CuI thermoelectric sensor is assessed according to the electrical conductivity of CuI and the thermoelectric response using the incorporated heater. Electrical conductivity provides the quality of a CuI layer. The electrical conductivity values in terms of S/m are 3977 for 0.8 μm CuI on bare glass, 414 for 1 μm CuI on rough glass, and 380 for 2 μm CuI on rough glass. A CuI layer on bare glass exhibits a low density of grain boundaries and interfacial defects, resulting in enhanced electrical conductivity by order of magnitude. Samples on a rough glass show similar electrical conductivities regardless of the CuI thickness. Figure 11a shows thermoelectric response sensitivity outcomes of 15.6 and 13.7 mV/K with 2 and 1 μm thick CuI layer on rough glass and 0.9 mV/K with 0.8 μm thick CuI layer on bare glass. The reduced electrical conductivity produces an enhanced thermopower.

The structural stability of the CuI layer on bare glass can be compromised during sample cleaning. A CuI layer with loose adhesion is likely to exhibit microcracks and deteriorated electric contacts that could bias the thermoelectric response. The present study increases the electrical resistivity of the CuI layer by employing a surface-modified glass to enable a higher density of defects and traps for charge carriers, supporting the development of a greater potential difference. A CuI layer on bare glass is unlikely to exhibit an enhanced density of defects, yet the electrical conductivity can be reduced for thin CuI layers.

The thermoelectric sensor is employed in several low-temperature sensing applications. Figure 11b depicts the sensing response to hydrogen gas from 0.1 to 4%, in which Pt/TiO_2_ catalytic ink is dispensed onto the heater region. The sensing test is conducted under ambient conditions with hydrogen in artificial air [27]. The sensor shows a response of 48 mV for 1% H_2_ under such a condition. Also, the time evolution of the voltage output is plotted for finger touch and for breath at the device center in Figure 11c,d, respectively. The thermoelectric sensor yields a result of 10–15 mV for the finger touch and 15–20 mV for breath, with both actions sustained for 3 s.

## 4. Discussion

### 4.1. Mechanism for Rough Soda-Lime Glass with Nanocavities

A rough glass specimen with nanocavities is developed by a conventional RIE process with a photoresist seed layer. The polymer layer enables roughening of the coated region and produces nanocavities from a porous layer of a hard residual photoresist. The residual layer produces a micromask effect that results in etching rate variations, and anisotropic etching is unlikely to occur due to the porous residual layer scattering the reactive ions [34]. Soda-lime glass has low purity and contains species that are less volatile during the RIE treatment, which can accumulate [35]. The etching of locally exposed glass, which is free of residuals, can produce nanocavities.

Characterization of the RIE-treated glass reveals the formation of non-volatile segregates. The residual hard layer consists of randomly distributed cone-shaped nanostructures on a porous ground layer with abundant cracks, as indicated in Figure 6a. The residual structures contain mainly NaF, which is in agreement with a previous study [35]. Thicker residual layers are obtained with a thicker photoresist seed layer, suggesting that the hard photoresist enhances the masking effect and surface roughening for the equivalent time for the RIE treatment. The resulting glass with nanocavities is important for the mechanical interlocking of supported layers featuring poor adhesion.

### 4.2. Effects of the Surface Roughness

Surface roughening produces abundant cracks in the supported layers, which could be beneficial when tuning the physical and chemical properties [22,36]. A rough substrate accelerates the iodination of the supported Cu layer due to the facile infiltration of iodine gas through the abundant grain boundaries. A rapid reaction can likely affect the crystallinity of CuI. Figure 10b shows enhanced crystallinity by 23% for (220) CuI on a rough glass specimen. CuI growth in the direction of the surface can be beneficial to reduce thermal conductivity [37].

### 4.3. Adhesion Strength of the CuI Layer

The deposition technique of the Cu seed material is likely to influence the adhesion strength of the converted CuI layer. In previous studies, cross-sectional images suggest that CuI derived from sputtered Cu is likely to have a stronger interfacial adhesion compared to that from evaporated Cu [38,39]. It is well known that the adhesion and film quality increase due to the highly energetic atmosphere produced by plasma-assisted deposition. CuI growth by iodination is effective on flexible substrates (cellulose paper or polymer films), showing good adhesion which can be attributed to surface pretreatment and surface roughness greater than bare glass substrate [21,40,41]. However, previous methods were limited to device fabrication on relatively large areas, as indicated in Table 1.

The direct deposition of a CuI layer is attainable in vacuum deposition systems. CuI shows good adhesion for plasma-assisted deposition [31]. Conversely, limitations in adhesion are visible for CuI layers prepared by thermal evaporation and pulsed laser deposition [45,46]. Thermally evaporated CuI is likely to exhibit reduced thermoelectric performance compared to the results after iodination methods [13,37]. Moreover, the scalable thickness of CuI is limited in vacuum systems.

**Figure 12:**
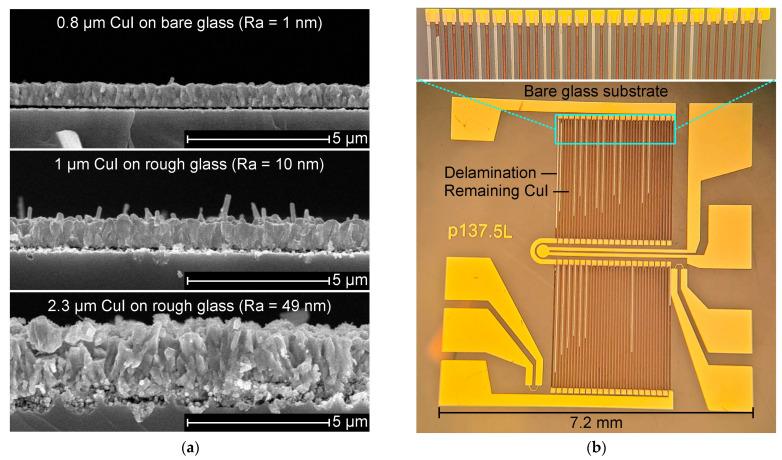
Delamination evaluation. (**a**) CuI layer on bare and rough glass specimens. (**b**) Delaminated CuI arms from the sensor layout on bare glass substrate.

A rough glass surface allows one readily to prepare CuI layer by iodination of a Cu layer, a promising approach to process relatively thick CuI films by a simple method. Figure 12a shows a 2.3 μm CuI layer prepared by the same process described in Section 2.2 with a Cu seed layer of 320 nm thick on a rough glass with Ra = 49 nm created with a photoresist layer of 16 um thick. The rough glass accommodates crystal growth into the nanocavities, enabling mechanical interlocking. CuI with good adhesion can withstand treatment in wet media and regular procedures involved in microfabrication based on photolithography.

A CuI layer of approximately 1 μm is evaluated to withstand a simple solvent-based cleaning process. The adhesion effectiveness was tested with 50 sensors on a 4𠌽 glass substrate according to the following steps. (1) Convert Cu into CuI, the final step in Section 2.2. (2) Clean by immersion while gently shaking in acetone and then in iso-propyl alcohol. (3) Heat-treat at 383 K for 3 min and cool to room temperature. (4) Rinse with DI water at a flow rate of 2 L/min. As a result of step (4), CuI arms were partially delaminated from every sensor on a bare glass substrate, as shown in Figure 12b, while the integrity of all sensors was preserved on a rough glass substrate.

### 4.4. Thermoelectric Response

The thermoelectric response is evaluated using the open-circuit voltage (V_oc_) of the thermoelectric sensor, which is the sensitivity parameter for sensor applications. The Seebeck coefficient or thermopower (S) of one p–n pair is 0.33 mV/K at room temperature, calculated from the slope between the voltage output and the temperature difference in Figure 11a and the formula ΔV = N × S × ΔT with N = 41 pairs [10]. The sensor shows enhanced thermopower compared to previous thermoelectric modules with CuI by at least 40%. Table 1 summarizes recent works involving thermoelectric modules with CuI, in which p–n pairs have relatively large footprints and yet yield lower thermopower levels. The proposed micro-thermoelectric sensor exhibits an enhanced open-circuit voltage and has the potential to be used in low-temperature energy harvesting and sensor applications.

## 5. Conclusions

A Cu layer converts into CuI by iodination, but it has the disadvantage of facile delamination from a bare glass substrate due to stress caused by significant material growth. This work proposes a rough glass substrate to support thermoelectric layers. A rough glass substrate is prepared by dry etching with a photoresist seed layer, resulting in a bumpy surface morphology and anchoring cavities of submicron size. The use of rough glass is a simple and effective method to enhance adhesion for CuI, resulting from vapor iodination under ambient conditions. A thermoelectric sensor with CuI/In-CoSb_3_ shows open-circuit voltages of 13.7 mV/K on rough glass and 0.9 mV/K on bare glass. A hydrogen sensor is prepared by depositing Pt/TiO_2_ catalyst onto the heater region, resulting in a sensitivity of 48 mV for 1% H_2_ under ambient conditions. The voltage output is in the range of 10~20 mV for finger touch and for breath in both cases for 3 s at the device center. As a result, an effective method is feasible to produce coarse glass with nanocavities, providing good anchoring and influencing the crystallinity of supported thermoelectric layers. CuI/In-CoSb_3_ on a rough substrate is more likely to be applied to body heat harvesting applications owing to the associated high thermovoltage and reduced area.

## Figures and Tables

**Table 1:** Thermopower of P–N Thermoelectric Modules at Room Temperature.

Material	Thickness [nm]	CuI Precursor	Area ^1^ [mm^2^]	Substrate	S ^1^ [mV/K]	Reference
CuI	300	Cu, I_2_(s)	-	PET	0.17	[21]
CuI	325	100 nm Cu, I_2_(s)	12 × 12	1000 μm glass	0.29	[13]
CuI/GZO	302/306	60 nm Cu, I_2_(s)	10 × 30	50 μm Kapton	0.09	[10]
CuI/Bi	1400/200	200 nm Cu, I_2_(g)	20 × 12	100 μm paper	0.18	[40]
CuI/ITO	550/-	CuI + I_2_ solution	45 × 20	ITO/polyimide	0.26	[42]
CuI/Monel ^2^	3/0.5 × 10^6^	CuSO_4_, NaI sol.	13 × 13	2.75 mm paper	0.23	[43]
P-N CoSb_3_	200/185	Ti, In, CoSb_3_	8 × 6	150 μm Kapton	0.34	[44]
CuI/In-CoSb_3_	1000/750	160 nm Cu, I_2_(g)	3 × 0.14	Soda-lime glass	0.33	This work

^1^ Normalized for a single p–n thermoelectric pair. ^2^ PEDOT:tosylate/CuI composite (p) and Monel wire (n).

## Data Availability

All relevant data are included in the main manuscript.
